# Release of VEGF from Dental Implant Improves Osteogenetic Process: Preliminary In Vitro Tests

**DOI:** 10.3390/ma10091052

**Published:** 2017-09-08

**Authors:** Barbara Zavan, Letizia Ferroni, Chiara Gardin, Stefano Sivolella, Adriano Piattelli, Eitan Mijiritsky

**Affiliations:** 1 Department of Biomedical Sciences, University of Padova, via G. Colombo 3, 35100 Padova, Italy; letizia.ferroni@unipd.it (L.F.); chiara.gardin@unipd.it (C.G.); 2 Maria Cecilia Hospital, GVM Care & Research, 48033 Cotignola, Ravenna, Italy; 3 Department of Neurosciences, University of Padova, via Giustiniani 5, 35100 Padova, Italy; stefano.sivolella@unipd.it; 4 Department of Medical, Oral, and Biotechnological Sciences, University of Chieti-Pescara, via dei Vestini 31, 66100 Chieti, Italy; adriano.piattelli@unich.it; 5 Department of Otolaryngology, Head and Neck and Maxillofacial Surgery, Sackler Faculty of Medicine, Tel-Aviv Sourasky Medical Center, Tel Aviv University, 6 Weitzman Street, 64239 Tel Aviv, Israel; mijiritsky@bezeqint.net

**Keywords:** biomimetic material, cell adhesion, osteogenesis, osseointegration, vascular endothelial growth factor

## Abstract

Introduction: During osseointegration process, the presence of an inflammatory event could negatively influence the proper osteogenetic ability of the implant surface. In order to reduce this possibility, an implementation of angiogenetic event through the release of Vascular Endothelial Growth Factor (VEGF) can be a tool as co-factor for osteoblastic differentiation. In this paper, novel dental implant surfaces enriched with VEGF have been tested. Material and methods: The ability of VEGF-enriched titanium implants to improve the osteogenetic properties of Mesenchymal stem cells (MSC), also in the presence of an inflammatory environment, have been in vitro tested. Molecular biology, morphological analyses, and biochemical tests have been performed in order to confirm biological properties of these surfaces. Results: Our results confirm that the presence of VEGF onto the implant surface is able not only to protect the cells from in vitro aging and from Reactive Oxygen Species (ROS) damage, but it also improves their osteogenic and endothelial differentiation, even in the presence of inflammatory cytokines. Conclusion: This study establishes a biologically powerful novel tool that could enhance bone repair in dental implant integration.

## 1. Introduction

A prerequisite for clinical success in dental implant surgery is implant osseointegration [1]. Several modifications of the implant surface, such as calcium-phosphate ceramic coatings, modification on macro/microporosity, or different surface treatments have been developed showing limited success in promoting integration [2,3,4]. Recent surface modification strategies directed at improving bone regeneration and osseointegration have involved the immobilization of Extracellular Matrix Components (ECM). This would facilitate the interaction with cellular integrin receptors, thus activating signaling pathways that promote osteoblastic differentiation and subsequent matrix mineralization [5,6]. The ECM proteins have therefore represented attractive targets for functionalizing surfaces of biomaterial thanks to their inherent bioactivity. Nevertheless, they have showed immunogenicity and complexities related to their purification and processing. Other strategies have focused their attention on short synthetic peptides that present the bioadhesive motif, such as peptides containing the arginine-glycine-aspartic acid (RGD) sequence, which mediates cell attachment to several matrix proteins, including vitronectin, fibronectin, bone sialoprotein, and osteopontin. However, these bio-inspired strategies have produced only marginal increases in implant osseointegration and mechanical fixation [7,8,9]. These disappointing results could find a reply in the lack of selectivity among integrins and, consequently, in non-discriminatory cell attachment.

In recent years, a novel therapeutic application has been based on the use of growth factors to improve regeneration of tissue since they are critical elements for new tissue production. In addition, in case of regenerative surgery they are able to perform feedback controls on inflammatory processes within the tissue graft [10,11]. Growth factors do not pass through a cell’s membrane, due to their ability to bind high-affinity cell receptors to take effect. Many growth factors are present in the ECM where they can be released during matrix degradation, thus acting during tissue remodeling and regeneration. In this context, several growth factors have been applied to bone regeneration strategies. One of the first used is Transforming Growth Factor-β (TGFβ) whose effect is highly variable and dependent on the type of cells and tissues. TGFβ regulates a broad range of cellular activities, such as cell proliferation, cell migration, cell differentiation, and ECM synthesis [12,13]. TGFβ promotes odontoblastic differentiation of Dental Pulp Stem Cells (DPSCs) and plays an important role in the immune response during the dental pulp injury [14]. Another growth factor used in bone regeneration is Bone Morphogenetic Protein (BMP) that belong to the TGFβ superfamily [15]. BMPs play important roles in the development and remodeling of the bone, promoting chemotaxis and aggregation of cells into osteogenic sites facilitating the differentiation of Mesenchymal Stem Cells (MSCs) into osteoblasts [16,17]. To date, more than 20 different forms of BMPs have been identified, and they are considered as the most potent growth factors promoting bone regeneration [18]. Other growth factors are used in regenerative medicine of the bone. These are represented by Fibroblast Growth Factor (FGF), which dramatically induces the mRNA expression of Dentin Sialophosphoprotein (DSPP) and Bone Sialoprotein (BSP) in immature adult rat incisor dental pulp cells [19,20], and Insulin-like Growth Factor (IGF), which contributes to odontogenesis and dental tissue repair by cell proliferation and differentiation [21,22]. In this context, we have focused our attention on Vascular Endothelial Growth Factor (VEGF). VEGF is a heparin-binding protein with particular affinity to endothelial cells and plays a key role in angiogenesis [23,24]. For a long time, VEGF has been mainly considered as a potent mitogenic factor for vascular endothelial cells, involved in the modulation of physiological angiogenesis, vascular permeability and also the occurrence/progression of tissue inflammation. More recently, VEGF has been recognized as a positive regulator of bone development, skeletal growth, and fracture repair, also stimulating proliferation and differentiation of bone-derived osteoblasts [25,26]. In addition, it has been reported that VEGF is involved in regulation of adipogenesis and osteogenesis in MSCs. VEGF is highly expressed in osteoblastic precursor cells and now it is known to stimulate bone formation [27]. Moreover, researchers tested the hypothesis that this growth factor could exert an important role as regulator of cell fate [28,29]. In particular, the authors suggest that VEGF could determine the differentiation that gives rise to osteoblasts or adipocytes. These results supported the model whereby VEGF regulates the commitment through an intracrine mechanism that is distinct from the role of secreted VEGF and its receptors.

In the present study, we have developed a titanium dental implant loaded with VEGF in order to improve osteogenesis during implant surgery. We hypothesized that coating titanium implants with this growth factor could enhance peri-implant bone formation and mechanical osseointegration, thus providing a simple, clinically relevant strategy for improving implant osseointegration.

## 2. Results

### 2.1. In Vitro Release of VEGF

**Figure 1:**
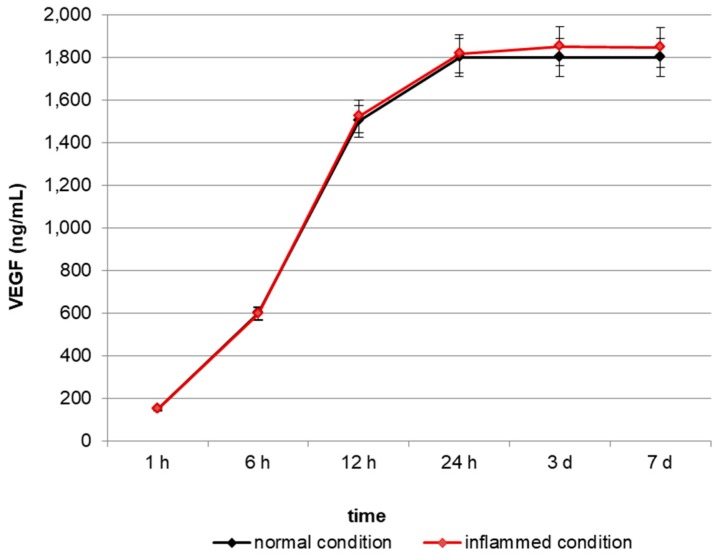
Quantification of Vascular Endothelial Growth Factor (VEGF) release in the cell culture medium. VEGF concentration increases in the medium in a time-dependent manner, reaching a plateau after 24 h.

In order to test the ability of VEGF-enriched dental implants to release VEGF in vitro, we quantified the growth factor in the cell culture medium up to 7 days by means of an ELISA test. As reported in Figure 1, VEGF concentration increases in the medium in a time-dependent manner, starting from a basal concentration of 180 ng/mL after 1 h and reaching the maximum level of 1800 ng/mL after 24 h.

### 2.2. Biocompatibility and Cell Proliferation

**Figure 2:**
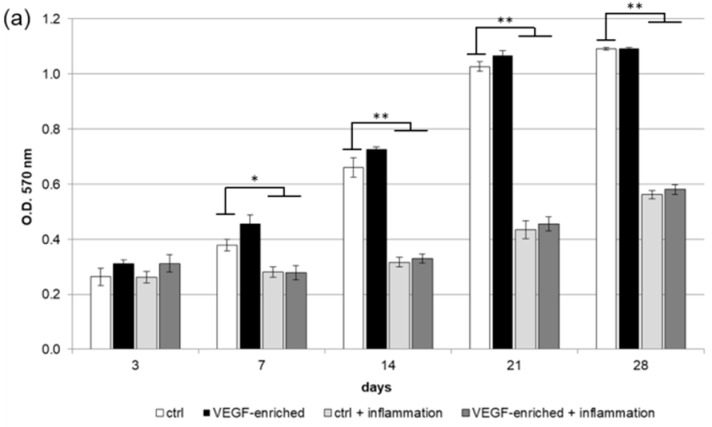

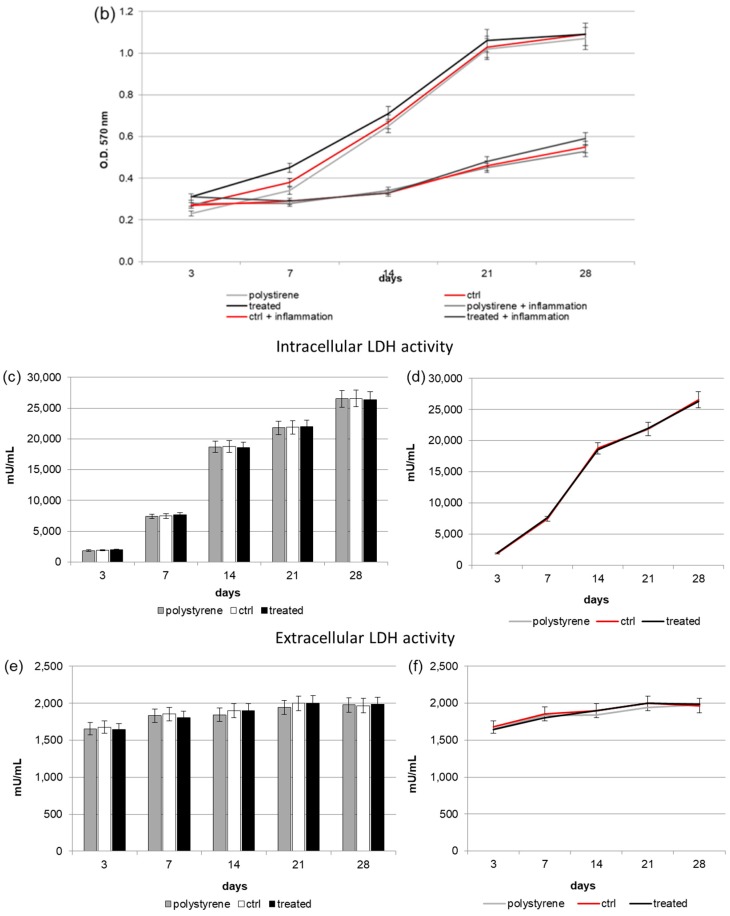
(**a**,**b**) 3-(4,5-Dimethylthiazol-2-yl)-2,5-Diphenyltetrazolium Bromide (MTT) assay. (**a**: in form of bars and **b**: in form of line). Cells are able to proliferate on both the control and VEGF-enriched surface from 3 to 28 days of culture with no statistical difference between the two surfaces. On the contrary, when cells are treated with Tumor Necrosis Factor α (TNFα) their proliferation is significantly reduced on both the control and the VEGF-enriched implant; (**c**,**d**) Quantification of intracellular Lactate Dehydrogenase (LDH) activity (**c**: in form of bars; **d**: in form of lines). Intracellular LDH activity proves that cells are able to produce metabolites if seeded onto both surfaces, with improved results after 7 days from seeding; (**e**,**f**) Quantification of extracellular LDH activity (**e**: in form of bars; **f**: in form of lines). Extracellular LDH activity confirms that metabolites are secreted by the cells and are not associated with damage of the membrane. Statistically significant differences are indicated as * *p* < 0.05, ** *p* < 0.01, and compared with the control condition.

The 3-(4,5-Dimethylthiazol-2-yl)-2,5-Diphenyltetrazolium Bromide (MTT) assay has been performed in order to test the implants’ biocompatibility. As shown in Figure 2a,b, human DPSCs were able to proliferate both on the control and VEGF-enriched implant surface with no statistical difference between the two surfaces. During culturing time, cells increase in number as demonstrated by the increasing value recorded. On the contrary, DPSCs treatment with the inflammatory cytokine Tumor Necrosis Factor alpha (TNFα) induces a significant decrease in cell proliferation both on the control and the VEGF-enriched surface.

### 2.3. Intracellular and Extracellular Lactate Dehydrogenase (LDH) Activity

Damage on cells surface has been evaluated with LDH activity assay. Figure 2c,d, related to intracellular LDH activity, shows that DPSCs were able to produce metabolites if seeded onto both surfaces, with improved results after 7 days from seeding. In Figure 2e,f, extracellular LDH activity is reported. Presence of LDH in extracellular compartment is generally correlated to a membrane damage, and this could be possibly due to the scaffold composition. The LDH activity measured in the culture medium confirms that there was no damage associated to the membrane. In addition, no difference between the two surfaces has been detected.

### 2.4. Reactive Oxygen Species (ROS) Production

**Figure 3:**
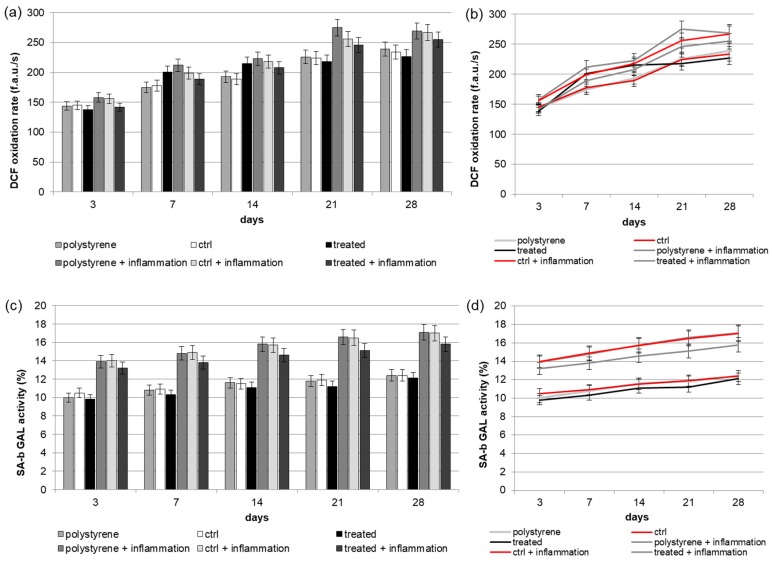
(**a**,**b**) (**a**: in form of bars and **b**: in form of line). Reactive Oxygen Species (ROS) production of Dental Pulp Stem Cells (DPSCs) seeded onto VEGF-enriched or control dental implants. Histograms show a slight time-dependent increase in metabolic activity in cells seeded onto both surfaces. The presence of VEGF on the implant surface is able to reduce the intracellular ROS production, especially under inflammatory conditions. Results are expressed as fluorescent arbitrary units per second (f.a.u./s); (**c**,**d**) (**c**: in form of bars and **d**: in form of line). Evaluation of Senescence-associated beta-galactosidase (SA-b GAL) (SA-b GAL) activity of DPSCs under normal or inflammatory conditions on surfaces combined or not with VEGF. Results show that cells cultured on implants loaded with VEGF have a lower SA-b GAL activity value compared to the cells seeded on the control one.

Analysis of intracellular redox equilibrium has been performed to test if VEGF released by the implant surface could induce oxidative stress, able to generate some modifications in signaling pathways. As reported in Figure 3a,b, a weak time-dependent increase in metabolic activity has been observed in cells seeded onto both surfaces. During culturing time, when DPSCs were seeded onto the VEGF-enriched dental implants, lower ROS level was recorded with respect to the value obtained from cells cultured onto control surfaces. Under inflammatory conditions, ROS production increased but, also in this case, the presence of VEGF onto the implants induced a small reduction in the measured ROS value.

### 2.5. Senescence

The presence of an inflammatory environment could affect normal cell growth, inducing the cells to enter in a state of irreversible arrested growth with altered functions. The senescence activity of stem cells following their in vitro culture has been evaluated by the β-galactosidase staining at selected time points of (3, 7, 14, 21 and 28 days), both in normal condition and in presence of inflammatory cytokines on surfaces enriched with VEGF or without it. As reported in Figure 3c,d, we observed an in vitro age-dependent increase in the Senescence-associated beta-galactosidase (SA-b GAL) activity. In particular, our results showed that cells cultured on implants containing VEGF had a lower SA-b GAL activity value compared to the cells cultured on the control ones. These results suggest that the aging effect on cells would be decreased by the VEGF treatment of dental implants.

### 2.6. Cell Morphology

**Figure 4:**
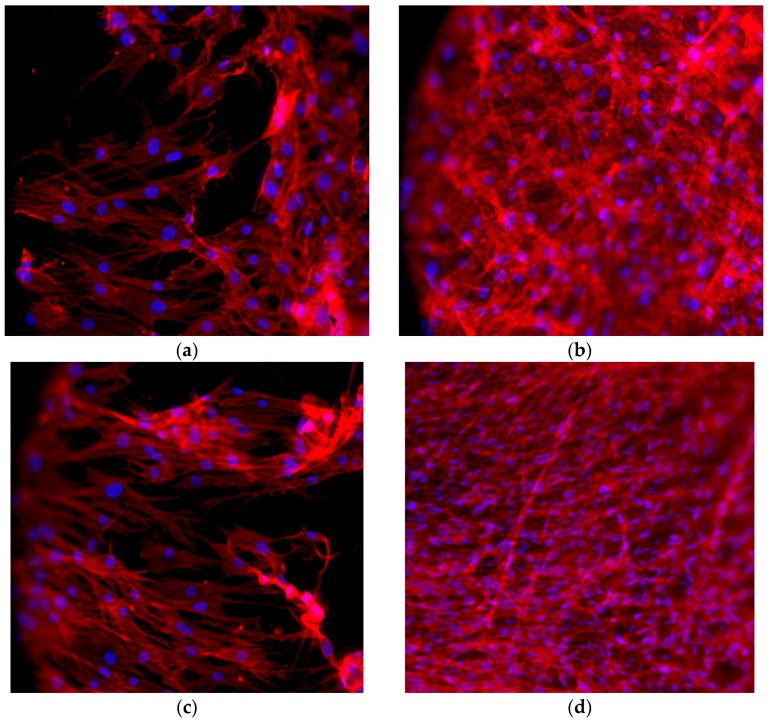
Immunofluorescence staining of the actin filament with phalloidin (in red). Cell nuclei are counterstained with Hoechst (in blue). (**a** for control; **b** on VEGF implants) After 7 days of culture, DPSCs have colonized the implant surface showing a star-like morphology; (**c** for control; **d** on VEGF implants) Cells are completely spread onto the implant surface after 14 days from seeding.

DPSCs were found to adhere to the dental implant surface, as revealed by the staining with the fluorescent phalloidin (Figure 4). After 7 days of culture, cells had colonized the implant surfaces and assumed the star-like morphology typically associated to an osteoblastic phenotype. After 14 days, cells were able to spread on all the implant surface.

**Figure 5:**
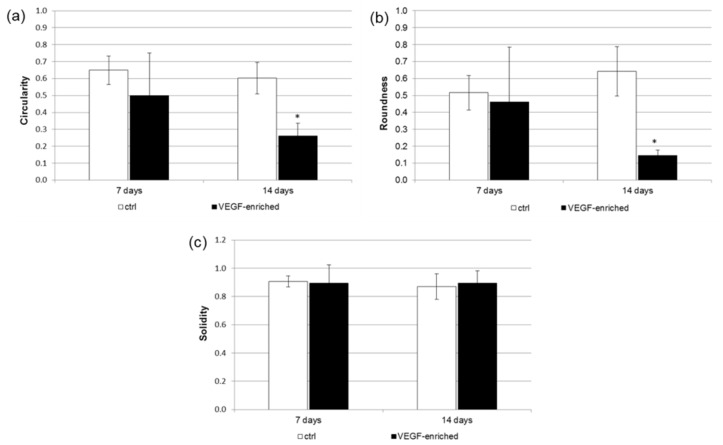
Analysis of the (**a**) Circularity; (**b**) Roundness; and (**c**) Solidity cell shape parameters after 7 and 14 days of culture onto the control and VEGF-enriched implants. * *p* < 0.05.

A detailed analysis of cell shape has additionally been performed. In particular, the Circularity, Roundness, and Solidity parameters have been considered. As shown in Figure 5, when cells were seeded onto the VEGF-enriched implants and cultured for 14 days, they assumed an elongated morphology, as suggested by the Circularity and Roundness calculated values. This would confirm that the cells lost the staminal rounded aspect for acquiring a more elongated morphology.

### 2.7. Effect of VEGF on Endothelial Cell Differentiation

**Figure 6:**
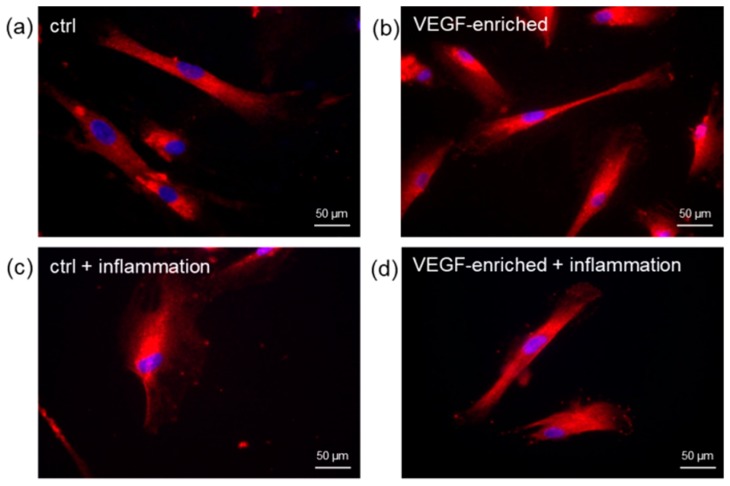

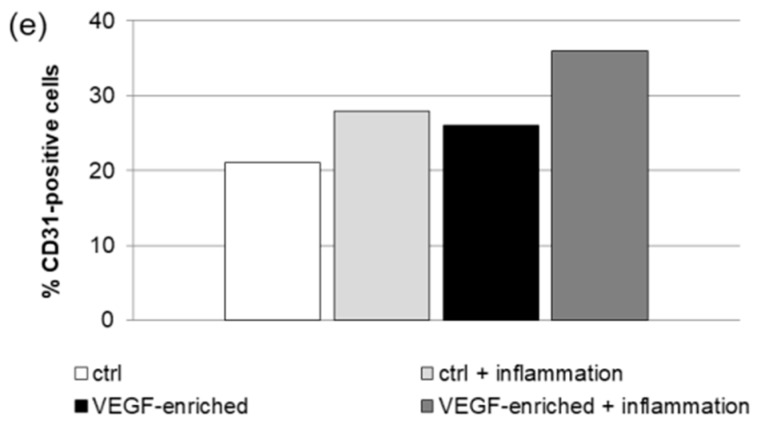
Immunofluorescence staining of the CD31 endothelial marker in DPSCs seeded onto (**a**) control implant; (**b**) implant loaded with VEGF; (**c**) control implant under inflammatory conditions; (**d**) VEGF-enriched implant under inflammatory conditions; (**e**) Evaluation of the % of CD31-positive cells.

To test the effect of VEGF present on the implant surface on endothelial differentiation of DPSCs, we have analyzed by immunofluorescence the percentage (%) of cells positive for the endothelial marker CD31 after 5 days of culture. As reported in Figure 6, the number of cells positive to CD31 increased when VEGF, released from the implants, was present in the cell environment (37% on VEGF-enriched implants vs. 28% onto control implants). The same trend was observed when cells were cultured under inflammatory conditions that could mimic the environment of several pathologies, such as peri-implantitis (29% on VEGF-enriched implants vs. 22% on control implants).

### 2.8. Osteogenic Commitment

The commitment of stem cells onto an osteoblastic phenotype has been evaluated by investigating the expression of several osteogenic markers, such as Alkaline phosphatase activity (ALP), Runx, osteopontin, osteonectin, osteocalcin, collagen type I, and endothelial markers, such as CD31, von Willebrand and VEGF by means of biochemical (for ALP) and with real-time PCR after 21 days of cultures. Tests were performed on cells seeded on implants loaded with VEGF or without it, both in normal condition and in presence of inflammatory cytokines.

**Figure 7:**
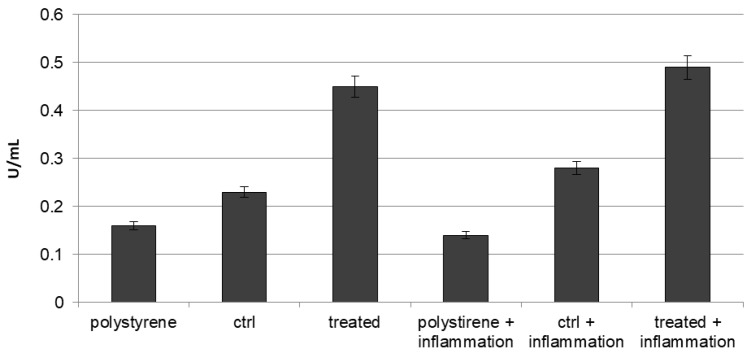
Alkaline phospahatase (ALP) production profiles in DPSCs seeded onto polystyrene, control or VEGF-enriched implants under normal or inflammatory conditions.

To confirm the early differentiation of cell towards osteoblast phenotype, the ALP activity (expressed as U/mL that is the amount of enzyme causing the hydrolysis of one micromole of pNPP per units per mL) was quantified into all cells condition. As reported on Figure 7, the extracellular ALP was higher in the VEGF-impregnated group than the controls (polystyrene and normal implant group), where it was lower (0.24 U/mL). To note, in all cases the presence of inflammatory condition induces an increase of ALP production.

**Figure 8:**
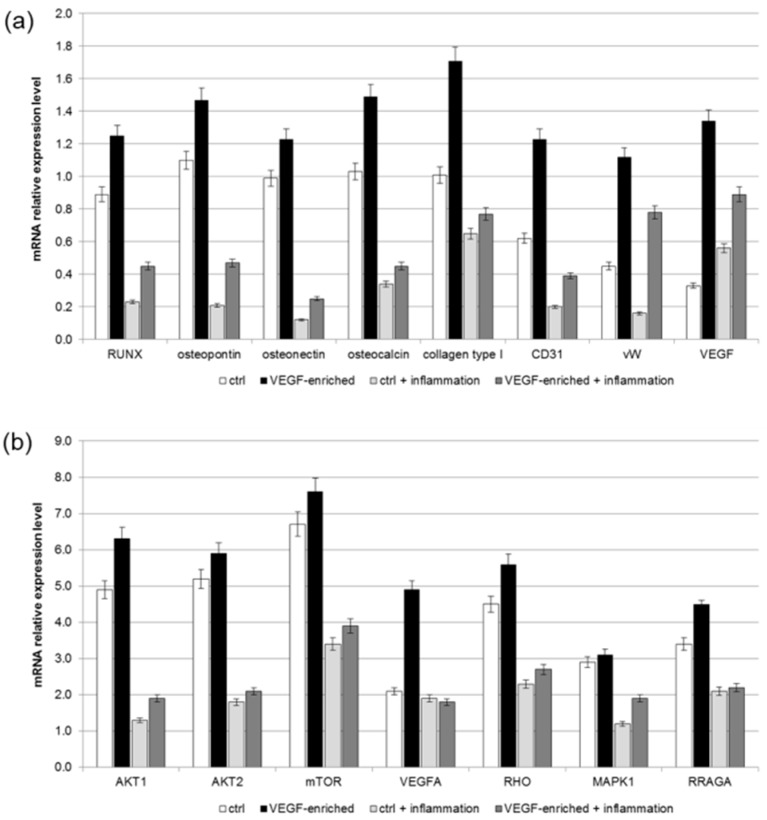
Gene expression profiles of (**a**) osteogenic and endothelial differentiation markers; (**b**) mTOR signaling pathway in DPSCs seeded onto control or VEGF-enriched implants under normal or inflammatory conditions. Values are expressed as 2∆∆Ct and normalized using DPSCs grown on tissue culture polystyrene as control.

As reported in Figure 8a, an increase in the expression of the osteogenic and endothelial markers was observed in all the conditions, thus confirming that the presence of VEGF exerts a positive effect on the differentiation process, even in the presence of an inflammatory environment.

In order to better understand the intracellular signaling pathways involved in the commitment of stem cells onto the osteogenic phenotype, a real-time PCR for the mTOR pathway markers has been performed. Results reported in Figure 8b show that, not only did the presence of VEGF improve the expression of this important factor, but it also induced an increase of its related proteins, such as AKT, MAPK1 and RRAGA. In addition, an increased expression has been detected for VEGF and Rho family of GTPases (RHO), which are involved in mechano-transduction functions. To note, the presence of VEGF on the implant’s surface improved the commitment of DPSCs onto the osteogenic phenotype even under inflammatory conditions.

## 3. Discussion

One of the principal factors inducing new tissue regeneration, i.e., when a dental implant is inserted into the bone or promoting tissue regeneration in the presence of damage, such as bone lysis process related to peri-implantitis, is vascularization. Clot formation and immune inflammatory responses are early biological events of peri-implant bone healing. Cells involved in such process in this microenvironment produce cytokines, and growth factors (i.e., PDGF, FGF; IGF; VEGF; BMP; IL1; TNF a needed to recruit osteogenic and vascular progenitor cells able to deposit extracellular matrix and its subsequent mineralization and to drive the process of neovascularization. To date, several strategies have been evaluated to improve bone regeneration under these conditions; however, little attention has been given to enhancing the vascularization around the implant [30,31,32,33,34,35]. The improvements of vascularization during bone regeneration related to use of enriched scaffolds are mainly correlated to the application of bone-like substitutes enriched on cells, growth factors of 3D structure. The use of VEGF on bone regenerations has been firmly studied in detail by Kempen [36], Kaigler [37], and Leach JK [38], that enriching different bone substitutes with VEGF confirmed that a high bone regeneration was present. In this view, no attention has ever been related to the enrichment of this growth factor on titanium surfaces.

In this study, a dental implant coated with VEGF has been designed and used as a platform to improve osteogenesis and angiogenesis. In this context, VEGF represents the natural stimulant, and DPSCs the cellular model. In detail, VEGF-enriched implants served as substrate to support cell adhesion, survival and differentiation. The MTT assay result was slightly higher for cells cultured on this type of implants compared to that of the unmodified implants on days 7–28; this was true also in the presence of inflammatory cytokines that usually are known to influence cell proliferation. In general, the MTT results show that the DPSCs grown on the VEGF-enriched implant exhibited higher proliferation rate than those seeded on the control one, both in normal and under inflammatory conditions. Interestingly, the presence of VEGF on the implant’s surface seems to protect the stem cells from senescence related to their in vitro aging process and to the presence of inflammatory cytokines. In our study, we additionally compared the endothelial differentiation of the cells cultured in presence of or without the inflammatory cytokine TNFα. Also in this case, the presence of VEGF on the dental implants actively improves the endothelial commitment of DPSCs, as demonstrated by the high percentage of CD31-positive cells. Lastly, the osteogenetic process has been evaluated by means of gene expression analyses. We observed that the interaction between VEGF-implant resulted in the upregulation of several osteogenic differentiation markers in DPSCs. Stem cells possess indeed the ability to differentiate into osteogenic cells when they are cultured under endothelial differentiation conditions.

## 4. Materials and Methods

### 4.1. Dental Implants

**Figure 9:**
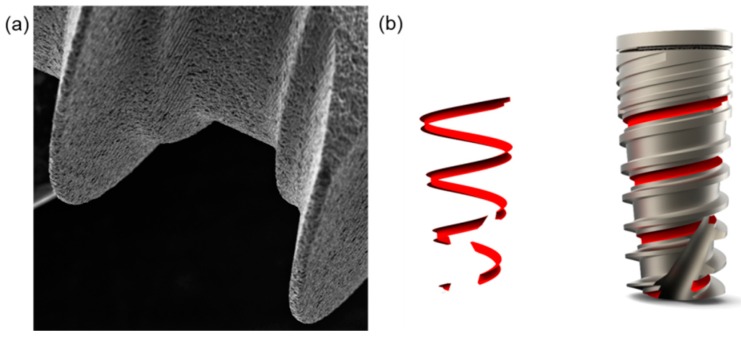
(**a**,**b**) Titanium dental implants used for the in vitro tests.

Dental implants were supplied by Ditron Dental (Ashkelon, Israel). In particular, MPI—Molecular Precision Implants with dimensions of 8 mm × 4.2 mm enriched with VEGF were used for the in vitro tests (Figure 9). The same implants without VEGF were used as control implants.

### 4.2. Quantitative Analysis of VEGF Release

VEGF release from titanium dental implant was measured in the culture medium at selected time points (1 h, 6 h, 12 h, 24 h, 3 days, 7 days) using the Human VEGF ELISA kit (Sigma-Aldrich, Saint Louis, MO, USA). We used 5 implants for each time point, results report the average of the different bathes value.

### 4.3. DPSCs Isolation and Seeding onto Dental Implant

Human dental pulps were extracted from healthy molar teeth of subjects, who had given written consent. Human DPSCs isolation was performed according to our previously published protocol [39]. The isolated cells were then cultured with Dulbecco’s Modified Eagle Medium (DMEM) (Lonza S.r.l., Milano, Italy) plus 10% Fetal Bovine Serum (FBS) (Bidachem S.p.A., Milano, Italy) and 1% Penicillin/Streptomycin (P/S) (EuroClone, Milan, Italy) to form complete DMEM (cDMEM). At confluence, cells have been detached from the culture plates using 0.25% trypsin (EuroClone, Milan, Italy), then seeded onto control and VEGF-enriched titanium implants at a density of 1 × 10^5^ cells per implant. For treatment under inflammatory conditions, cells were cultured on both types of implants in presence of 10 ng/mL of TNFα (Miltenyi Biotec, Bergisch Gladbach, Germany). Cells were maintained in culture up to 28 days, changing the medium twice a week.

### 4.4. MTT Assay

To determine the proliferation rate of cells grown onto control and VEGF-enriched implants, the MTT (methyl thiazolyl-tetrazolium)-based cytotoxicity assay was performed according to the method of Denizot and Lang with minor modifications [40,41]. The test is based on mitochondrial viability, i.e., only functional mitochondria can oxidize an MTT solution, giving a typical blue–violet end-product. After harvesting the culture medium, the cells were incubated for 3 h at 37 °C in 1 mL of 0.5 mg/mL MTT solution prepared in Phosphate Buffer Saline (PBS, EuroClone) solution. After removal of the MTT solution by pipette, 0.5 mL of 10% dimethyl sulfoxide in isopropanol (iDMSO) was added for 30 min at 37 °C. For each sample, absorbance values at 570 nm were recorded in duplicate on 200 μL aliquots deposited in 96-well plates using a multilabel plate reader (Victor 3, Perkin Elmer, Waltham, MA, USA). All samples were examined after 3, 7, 14, 21 and 28 days from seeding.

### 4.5. LDH Activity

LDH activity was measured using a specific LDH Activity Assay Kit (Sigma-Aldrich) at 3, 7, 14, 21 and 28 days of cell culture. All conditions were tested in duplicate. The culture medium was reserved to determine extracellular LDH activity. The intracellular LDH activity was estimated after cells lysis with the assay buffer contained in the kit. All samples were incubated with a supplied reaction mixture, resulting in a product whose absorbance was measured at 450 nm using Victor 3 plate reader.

### 4.6. ROS Measurements

The OxiSelect™ ROS Assay Kit (Cell Biolabs, Selangor, Malaysia) was used for measuring intracellular ROS activity. The assay employs the fluorogenic probe DCFH-DA, which is cell permeant. After deacetylation by cellular esterases, the probe is trapped as the non-fluorescent DCFH. Inside the cell, DCFH reacts with intracellular ROS to form the fluorescent product DCF. The fluorescence was recorded on the Victor 3 plate reader at 530 nm.

### 4.7. SA-b GAL Staining

Beta-galactosidase staining was performed using the Senescence-associated β-galactosidase staining kit (Cell Signaling Technology, Danvers, MA, USA). DSPCs were fixed with a fixative solution, and stained with the β-Galactosidase staining solution at 37 °C O/N. The number of positive and negative cells were then counted in five random fields under the microscope, and the percentage of SA-b GAL positive cells was calculated as the number of positive cells divided by the total number of cells counted.

### 4.8. Cell Shape Analysis

Cell shape was evaluated on samples stained for 40 min with 5 mg/mL phalloidin. Briefly, cells were fixed in 4% paraformaldehyde in PBS for 10 min, then permeabilized with 0.1% triton X-100 (Sigma-Aldrich, Saint Louis, MA, USA) in PBS for 30 min at room temperature. Phalloidin was then used for fluorescent staining of actin filaments, whilst nuclear staining was performed with 2 μg/mL Hoechst H33342 (Sigma-Aldrich) solution for 5 min. Images were acquired with the inverted optical microscope DMI4000 B (Leica Microsystems, Wetzlar, Germany). Then, ImageJ software (http://rsb.info.nih.gov/ij) was used to calculate cell area and different shape descriptors of at least 30 distinct cells. In detail, the Circularity (C), Roundness (R), and Solidity (S) of cells were calculated according to the following equations [42]:

Circularity = C = 4πA/P^2^ (1)

where A is the cell area and P is the perimeter;

Roundness = R = a/b(2)

where a and b are the width and length of the minimum bounding, respectively;

Solidity = S = A/ConvexA(3)

where ConvexA is the area enclosed by the smallest shell that borders all the points of the cell.

### 4.9. Immunofluorescence

Cells were fixed in 4% paraformaldehyde in PBS for 10 min, then incubated in 2% Bovine Serum Albumin (BSA, Sigma-Aldrich) in PBS for 30 min at room temperature. Cells were incubated with the rabbit polyclonal anti-human CD31 antibody (Abcam, Cambridge, UK) in 2% BSA solution in a humidified chamber at 4 °C O/N. Immunofluorescence staining was performed using the secondary antibody DyLight 549-labeled anti-rabbit IgG (H + L) (KPL, Gaithersburg, MD, USA) diluted 1/1000 in 2% BSA for 1 h at room temperature. Nuclear staining was performed with 2 μg/mL Hoechst H33342 solution for 5 min.

The percent of differentiated cells was calculated by counting the positive cells for CD31 compared to the total number of cells present on the surface.

### 4.10. Real-Time RT-PCR

Total RNA was extracted from the DPSCs seeded onto VEGF-enriched or control dental implants with the RNeasy Mini Kit (Qiagen GmbH, Hilden, Germany), including DNase digestion with the RNase-Free DNase Set (Qiagen). For the first-strand cDNA synthesis, 500 ng of total RNA of each sample was reverse-transcribed with the SensiFAST™ cDNA Synthesis Kit (Bioline, London, UK), following the manufacturer’s protocol. Human primers were selected for each target gene using the Primer 3 software. Real-time PCRs were run using the chosen primers at a concentration of 400 nM and SensiFAST™ SYBR No-ROX Kit (Bioline) on a Rotor-Gene 3000 (Corbett Research, Sydney, NSW, Australia). The thermal cycling conditions were as follows: 2 min denaturation at 95 °C; 40 cycles of 5 s denaturation at 95 °C; annealing for 10 s at 60 °C; and 20 s elongation at 72 °C. Differences in gene expression were assessed with the 2∆∆Ct method [43] using DPSCs cultured in cDMEM on tissue culture polystyrene as a control. Values were normalized to the expression of the Transferrin Receptor (TFRC) internal reference, whose abundance did not change under our experimental conditions. All experiments were conducted using three different preparations and repeated three times.

### 4.11. Statistical Analyses

The mean values for quantitative data were compared applying non-parametric Kruskal–Wallis test for RT-PCR results. T tests were used to determine significant differences (*p*, 0.05). Repeatability was calculated as the standard deviation of the difference between measurements. All testing was performed in SPSS 16.0 software (SPSS Inc., Chicago, IL, USA) (license of the University of Padua, Italy). Each test has been performed on 5 different implants for each time point and repeated 3 times.

### 4.12. ALP Activity Measurements

The alkaline phosphatase (ALP) activity was measured after 3 weeks of cell culture to evaluate the initial differentiation of MSC into preosteoblasts. Abcam’s Alkaline phosphates kit (colorimetric) has been used to detect the intracellular and extracellular ALP activity. The kit uses p-nitrophenyl phosphate (pNPP) as a phosphatase substrate which adsorbed at 405 nm when dephosphorylated by ALP. According to the manufacturer protocol, the culture medium from each sample group was collected and pooled together. At the same time, cells were washed with PBS and then homogenized with ALP Assay Buffer (300 μL in total for each group) and centrifuged at 13,000 rpm for 3 min to remove insoluble material. Different volumes of samples (medium and cells) were then added into 96-well plates, bringing the total volume in each well up to 80 μL with Assay Buffer; 80 μL of fresh medium was also utilized as sample background control.

Thereafter, 50 μL of 5 mM pNPP solution was added to each well containing test samples and background control and incubated for 60 min at 25 °C, protecting the plate from the light. A standard curve of 0, 4, 6, 12, 16 and 20 nmol/well was generated from 1 mM pNPP standard solution bringing the final volume to 120 μL with Assay Buffer. All reactions were then stopped by adding 20 μL of Stop solution into each standard and sample reaction except the sample background control reaction. Optical density was read at 405 nm in a microplate reader (Victor). The results were normalized subtracting the value derived from the zero standards from all standards, samples and sample background control. The pNP standard curve was plotted to identify the pNP concentration in each sample. ALP activity of the test samples was calculated as follow:

ALP activity (U/mL) = A/V/T (4)

where A is the amount of pNP generated by samples (in μmol). V is the amount of sample added in the assay well (in mL). T is the reaction times (in minutes).

## 5. Conclusions

In conclusion, we have presented herein a novel titanium dental implant designed and manufactured to promote vascularization. The results of our morphological, biochemical and biological in vitro tests showed that the implant loaded with VEGF supported the growth and differentiation of DPSCs also in the presence of an inflammatory environment. Although additional in vivo analyses would lead to more positive conclusions, this VEGF-enriched implant could represent a good foothold for improving the vascularization of dental implants also in cases of oral pathologies, such as peri-implantitis. For this reason, in the future this novel developed implant could have great potential for in vivo applications.
